# Sinfonevada: Dataset of Floristic diversity in Sierra Nevada forests (SE Spain)

**DOI:** 10.3897/phytokeys.35.6363

**Published:** 2014-02-17

**Authors:** Antonio Jesús Pérez-Luque, Francisco Javier Bonet, Ramón Pérez-Pérez, Rut Aspizua, Juan Lorite, Regino Zamora

**Affiliations:** 1 Laboratorio de Ecología (iEcolab), Instituto Interuniversitario de Investigación del Sistema Tierra en Andalucía (CEAMA), Universidad de Granada, Avenida del Mediterráneo s/n, 18006, Granada, Spain; 2 Grupo de Ecología Terrestre, Departamento de Ecología, Universidad de Granada, Facultad de Ciencias, Campus de Fuentenueva s/n, 18071, Granada, Spain; 3 Agencia de Medio Ambiente y Agua de Andalucía. Consejería de Medio Ambiente y Ordenación del Territorio. Junta de Andalucía, C/ Joaquina Egüaras, 10, 18003, Granada, Spain; 4 Departamento de Botánica, Universidad de Granada, Facultad de Ciencias, Campus de Fuentenueva s/n, 18071, Granada, Spain

**Keywords:** Sierra Nevada, Spain, floristic inventories, vascular plant, Liliopsida, Magnoliopsida, global-change monitoring, occurrence, observation

## Abstract

The Sinfonevada database is a forest inventory that contains information on the forest ecosystem in the Sierra Nevada mountains (SE Spain). The Sinfonevada dataset contains more than 7,500 occurrence records belonging to 270 taxa (24 of these threatened) from floristic inventories of the Sinfonevada Forest inventory. Expert field workers collected the information. The whole dataset underwent a quality control by botanists with broad expertise in Sierra Nevada flora. This floristic inventory was created to gather useful information for the proper management of *Pinus* plantations in Sierra Nevada. This is the only dataset that shows a comprehensive view of the forest flora in Sierra Nevada. This is the reason why it is being used to assess the biodiversity in the very dense pine plantations on this massif. With this dataset, managers have improved their ability to decide where to apply forest treatments in order to avoid biodiversity loss. The dataset forms part of the Sierra Nevada Global Change Observatory (OBSNEV), a long-term research project designed to compile socio-ecological information on the major ecosystem types in order to identify the impacts of global change in this area.

## Project details

### Project title

Sierra Nevada Global Change Observatory (OBSNEV)

### Personnel

Regino Jesús Zamora Rodríguez (Principal Investigator)

### Funding

All the information contained in Sinfonevada was gathered by TRAGSA (Transformación Agraria S.A.), a public company funded by the Spanish Ministry of the Environment. The Sierra Nevada Global Change Observatory is funded by the Andalusian Regional Government (via Environmental Protection Agency) and by the Spanish Government (via “Fundación Biodiversidad”, which is a Public Foundation).

### Study area descriptions/descriptor

**Figure 1.**
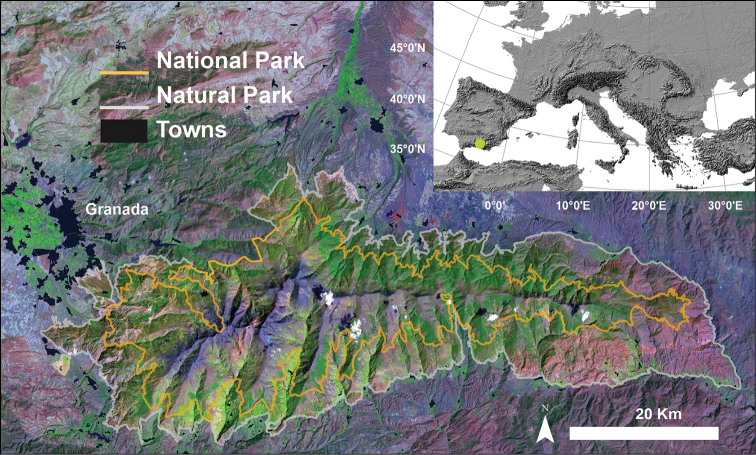
Location of Sierra Nevada mountain (southern Spain). The boundaries of the National and Natural Parks are shown. We used a Landsat 5 image (2001) as background.

Sierra Nevada (Andalusia, SE Spain), is a mountainous region with an altitudinal range between 860 m and 3482 m a.s.l. covering more than 2000 km^2^ (Figure 1). The climate is Mediterranean, characterized by cold winters and hot summers, with pronounced summer drought (July-August). The annual average temperature decreases in altitude from 12–16°C below 1500 m to 0°C above 3000 m a.s.l., and the annual average precipitation is about 600 mm. Additionally, the complex orography of the mountains causes strong climatic contrasts between the sunny, dry south-facing slopes and the shaded, wetter north-facing slopes. Annual precipitation ranges from less than 250 mm in the lowest parts of the mountain range to more than 700 mm in the summit areas. Winter precipitation is mainly in the form of snow above 2000 m of altitude. The Sierra Nevada mountain range hosts a high number of endemic plant species (c. 80; Lorite et al. 2007) for a total of 2,100 species of vascular plants (25% and 20% of Spanish and European flora, respectively), being considered one of the most important biodiversity hotspots in the Mediterranean region (Blanca et al. 1998).

This mountain range has several legal protections: Biosphere Reserve MAB Committee UNESCO; Special Protection Area and Site of Community Importance (Natura 2000 network); and National Park. The area includes 61 municipalities with more than 90,000 inhabitants. The main economic activities are agriculture, tourism, cattle raising, beekeeping, mining, and skiing (Bonet el al. 2010).

### Design description

Sierra Nevada Global Change Observatory (OBSNEV) (Bonet et al. 2011) is a long-term research project which is being undertaken at Sierra Nevada Biosphere Reserve (SE Spain). It is intended to compile the information necessary for identifying as early as possible the impacts of global change, in order to design management mechanisms to minimize these impacts and adapt the system to new scenarios (Aspizua et al. 2010, Bonet el al. 2010). The general objectives are to:

• Evaluate the functioning of ecosystems in the Sierra Nevada Nature Reserve, their natural processes and dynamics over a medium-term timescale.

• Identify population dynamics, phenological changes, and conservation issues regarding key species that could be considered indicators of ecological processes.

• Identify the impact of global change on monitored species, ecosystems, and natural resources, providing an overview of trends of change that could help foster ecosystem resilience.

• Design mechanisms to assess the effectiveness and efficiency of management activities performed in the Sierra Nevada in order to implement an adaptive management framework.

• Help to disseminate information of general interest concerning the values and importance of Sierra Nevada.

**Figure 2.**
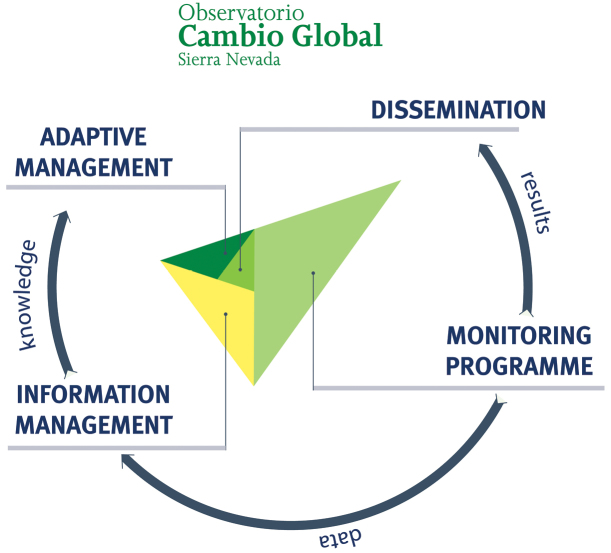
Structure of the Sierra Nevada Global Change Observatory. The four cornerstones of the research project are shown: monitoring program, adaptive management, information systems and dissemination. A monitoring program is needed to identify the impacts of global change over Sierra Nevada. The information compiled needs to be transformed into useful knowledge for the managers to carry out an active and adaptive management of natural resources. To achieve this, it is essential that all data be integrated and analysed in an information system. Finally, the general public should be informed of both the results obtained and methodologies used, through effective outreach activities.

The Sierra Nevada Global Change Observatory has four cornerstones (Figure 2): 1) a monitoring program with 40 methodologies that collect information on ecosystem functioning; 2) an information system to store and manage all the information gathered; 3) a plan to promote adaptive management of natural resources using the knowledge amassed through the monitoring programme; and 4) an outreach program to disseminate all the available information to potential users.

The Sierra Nevada Global Change Observatory is linked to other national (Zamora and Bonet 2011) and international monitoring networks: GLOCHAMORE (Global Change in Mountain Regions) (Björnsen 2005), GLOCHAMOST (Global Change in Mountain Sites) (Schaaf 2009), LTER-Spain (Long-Term Ecological Research).

Sierra Nevada Global Change Observatory is collecting socio-ecological information on the major ecosystem types found in Sierra Nevada. This information is being integrated in an Information System (http://obsnev.es/linaria.html - Pérez-Pérez et al. 2012. (Free access upon registration). The dataset described here is a good example of this idea. We have created a relational database to store the floristic inventories prepared in 2004–2005. Thanks to this work, all this valuable and unique information will be available to scientists and environmental managers worldwide.

## Data published through

GBIF: http://www.gbif.es:8080/ipt/resource.do?r=sinfonevada

## Taxonomic coverage

### General taxonomic coverage description

**Figure 3.**
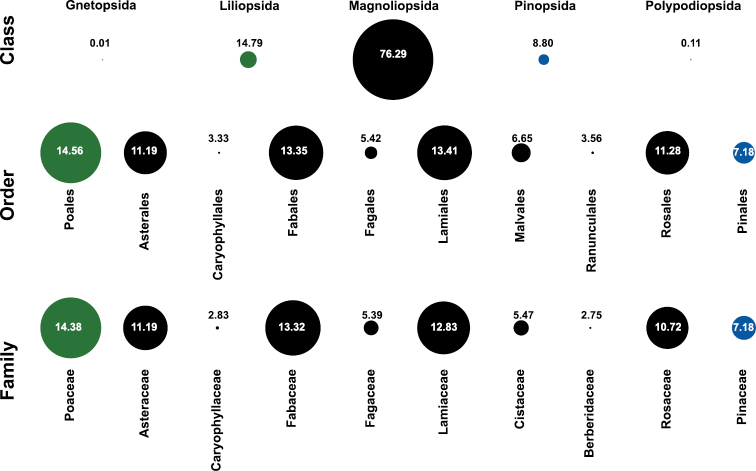
Taxonomic coverage. The figure shows the taxonomic coverage for class, order and family. The circles size are proportional to the number of records in the Sinfonevada database. Numbers indicates the percentage of records. All taxonomic classes included in the database are shown. For order and family rank, only the top 10 are shown. Colour indicates the taxonomic class: green (Liliopsida); black (Magnoliopsida) and blue (Pinopsida).

Most of the species recorded in the inventories belong to class Magnoliopsida (6,042 records; 76.28 %) and Liliopsida (1,171 records; 14.78 %). The top 10 of the orders (Figure 3) include Poales (1153 records; 14.56 %) for the class Liliopsida, Lamiales (1062 records; 13.41 %) for Magnoliopsida and Pinales (569 records; 7.18 %). In these collection, 57 families are represented, with Poaceae, Fabaceae, and Lamiaceae being the families with major number of records (Table 1) (Figure 3). The collection includes 270 taxa belonging to 159 genera, *Pinus* and *Thymus* being the most represented ones in the database. There are 24 threatened taxa (Table 2).

**Table 1.** The top 20 of the families represented in the collection.

Family	records	%
Poaceae	1139	14.38
Fabaceae	1055	13.32
Lamiaceae	1016	12.83
Asteraceae	886	11.19
Rosaceae	849	10.72
Pinaceae	569	7.18
Cistaceae	433	5.47
Fagaceae	427	5.39
Caryophyllaceae	224	2.83
Berberidaceae	218	2.75
Apiaceae	160	2.02
Cupressaceae	128	1.62
Thymelaeaceae	94	1.19
Rubiaceae	89	1.12
Brassicaceae	87	1.10
Crassulaceae	78	0.98
Euphorbiaceae	65	0.82
Ranunculaceae	64	0.81
Scrophulariaceae	38	0.48
Rhamnaceae	34	0.43

**Table 2.** Threatened species included in SINFONEVADA dataset.

Scientific Name	Bern ^a^	CITES^b^	Habitat Directive ^c^	Spanish Red List ^d^	Andalusian Red List ^e^	IUCN^f^
*Acer monspessulanum* L.					NT	VU
*Acer opalus* subsp. *granatense* (Boiss.) Font Quer & Rothm.					NT	VU
*Amelanchier ovalis* Medik.					NT	LR-lc
*Armeria filicaulis* Boiss. subsp. *nevadensis* Nieto Fel., Rosselló & Fuertes				VU	VU	VU
*Celtis australis* A.Rich.					NT	LR-lc
*Centaurea bombycina* Boiss. subsp. *bombycina*				VU	VU	VU
*Centaurea monticola* Boiss. ex DC.				VU	VU	VU
*Centaurea pulvinata* (Blanca) Blanca	Appendix I		Annex II	VU	VU	VU
*Cephalanthera longifolia* (L.) Fritsch		Annex B				
*Cotoneaster granatensis* Boiss.					NT	VU
*Cytisus galianoi* Talavera & P.E.Gibbs					NT	
*Erica terminalis* Klotzsch					NT	VU
*Euphorbia nevadensis* Boiss. & Reut.			Annex IV		NT	LR-nt
*Pinus sylvestris* L. var. *nevadensis* Christ				EN	EN	VU
*Potentilla reuteri* Boiss.				NT	NT	VU
*Prunus avium* (L.) L.						VU
*Prunus mahaleb* L.						VU
*Prunus ramburii* Boiss.				VU	VU	
*Quercus pyrenaica* Willd.					NT	LR-cd
*Reseda complicata* Bory				VU	VU	
*Salix caprea* L.					EN	EN
*Salix eleagnos* subsp. *angustifolia* (Cariot) Rech. f.						LR-cd
*Santolina elegans* Boiss. ex DC.			Annex IV	VU	VU	VU
*Sorbus aria* Wimm. ex Nyman					NT	VU

**^a^** Bern: Convention on the Conservation of European Wildlife and Natural Habitats (Bern Convention). **^b^** CITES: Convention on International Trade in Endangered Species of Wild Fauna and Flora. Species included in its. **^c^** Species included in the Habitat Directive Annex (EC 1992) **^d^** 2010 Red List of Spanish vascular flora (Moreno 2010) **^e^** 2005 Red List of vascular flora of Andalusia (Cabezudo et al. 2005) **^f^** IUCN category in Sierra Nevada (IUCN 2001, Blanca et al. 1998, Blanca et al. 2001, Lorite et al. 2007) *EN:* Endangered; *VU:* Vulnerable; *NT:* Near threatened; *LR-nt:* Lower Risk-Near Threatened; *LR-cd:* Lower Risk-Conservation Dependent; *LR-lc:* Lower Risk-Least Concern

## Taxonomic ranks

**Kingdom:** Plantae

**Phylum:** Pteridophyta, Spermatophyta

**Class:** Gnetopsida, Liliopsida (Monocotyledones), Magnoliopsida (Dicotyledones), Pinopsida, Polypodiopsida

**Order:** Apiales, Asparagales, Asterales, Brassicales, Caryophyllales, Cucurbitales, Cupressales, Dipsacales, Ephedrales, Ericales, Fabales, Fagales, Gentianales, Geraniales, Lamiales, Liliales, Malpighiales, Malvales, Pinales, Poales, Polypodiales, Ranunculales, Rosales, Santalales, Sapindales, Saxifragales, Solanales, Umbellales

**Family:** Amaryllidaceae, Anacardiaceae, Apiaceae, Apocynaceae, Araliaceae, Asparagaceae, Asteraceae, Berberidaceae, Brassicaceae, Capparaceae, Caprifoliaceae, Caryophyllaceae, Cistaceae, Clusiaceae, Colchicaceae, Convolvulaceae, Coriariaceae, Crassulaceae, Cupressaceae, Cyperaceae, Dennstaedtiaceae, Dipsacaceae, Ephedraceae, Ericaceae, Euphorbiaceae, Fabaceae, Fagaceae, Geraniaceae, Iridaceae, Juglandaceae, Juncaceae, Lamiaceae, Leguminosae, Oleaceae, Orchidaceae, Paeoniaceae, Pinaceae, Plantaginaceae, Plumbaginaceae, Poaceae, Polygonaceae, Ranunculaceae, Resedaceae, Rhamnaceae, Rosaceae, Rubiaceae, Rutaceae, Salicaceae, Santalaceae, Sapindaceae, Scrophulariaceae, Smilacaceae, Thymelaeaceae, Ulmaceae, Umbelliferae, Urticaceae, Violaceae

## Spatial coverage

### General spatial coverage

The SINFONEVADA forest inventory was conducted in the main forests of Sierra Nevada mountainous region (Figure 1) (for a description of Sierra Nevada see study area of the Project section). The main forest units of Sierra Nevada (Figure 5) are pine plantations (*Pinus halepensis* Mill., *Pinus pinaster* Ait., *Pinus nigra* Arnold subsp. *salzmannii* (Dunal) Franco, and *Pinus sylvestris* L.), evergreen holm oak *Quercus ilex* subsp. *ballota* (Desf.) Samp forests, deciduous broadleaf forests (*Quercus pyrenaica* Willd, *Acer opalus* subsp. *granatense* (Boiss.) Font Quer & Rothm., *Sorbus aria* (L.) Crantz), and autochthonous pine *Pinus sylvestris* L. var. *nevadensis* Christ forests.

### Coordinates

36°52'12"N and 37°15'36"N Latitude; 3°41'24"W and 2°33'36"W Longitude

### Temporal coverage

2004–2005

## Natural collections description

**Parent collection identifier:** NA

**Collection name:** Sinfonevada: Dataset of floristic diversity in Sierra Nevada forest (SE Spain)

### Collection identifier

http://www.gbif.es:8080/ipt/manage/metadata-collections.do?r=sinfonevada

## Methods

### Method step description

This inventory was undertaken in 2004 and the database generated contains information relative to forest attributes and occurrence data (see below). This information, originally stored in a Microsoft Access database, has been integrated into the project’s information system.

**Study extent description:** The floristic inventories were conducted at the main forest units of the Sierra Nevada (Andalusia, SE Spain). Forest cover in Sierra Nevada is dominated by pine plantations (*Pinus halepensis* Mill., *Pinus pinaster* Ait., *Pinus nigra* Arnold subsp. *salzmannii* (Dunal) Franco, and *Pinus sylvestris* L.) covering approximately 40,000 ha. Most of them were planted in the period 1960–1980. The main native forests of Sierra Nevada are dominated by the evergreen holm oak *Quercus ilex* subsp. *ballota* (Desf.) Samp. occupying low and medium mountain areas (8,800 ha.) and Pyrenean oak *Quercus pyrenaica* Willd ranging from 1,100 to 2,000 m a.s.l., covering about 2,000 ha. Autochthonous pine *Pinus sylvestris* var. *nevadensis* forests can also be found in small patches at high altitudes with a characteristically low tree cover.

**Sampling description:** SINFONEVADA Forest Inventory was established over an extensive network of 600 long-term permanent plots distributed within the main forest units of the Sierra Nevada: pine plantations, evergreen *Quercus ilex* forests, and deciduous broadleaf forests. The network of plots is a random sample stratiﬁed by land cover and altitude, covering a gradient of 974–2439 m a.s.l. (Figure 5).

Each inventory plot has three sampling units: i) a forest inventory plot (20 × 20 m); ii) a 5-m radius subplot for the estimation of the regeneration; iii) and a 10-m radius subplot for species composition and abundance.

Each live tree with a diameter at breast height (dbh) > 7.5 cm was tallied by species and dbh in the forest inventory plot. This information was used to calculate forest attributes (tree basal area, tree volume, canopy cover). The regeneration was measured in the 5-m radius subplot (78.5 m^2^ in area) as seedling abundance of the main tree species.

The species composition and diversity was recorded within a 10-m radius subplot (314 m^2^ in area) using the Braun-Blanquet cover-abundance scale (Braun-Blanquet 1964).

### Quality control description

Prior to the storing of this information in the database, all the data were assessed by a quality-control process. Each sampling plot was checked to ensure that the geographical coordinates were correct. We used the databases of International Plant Names Index (IPNI 2013) and Catalogue of Life/Species 2000 (Roskov et al. 2013) to verify the taxonomical classification. The specimens were taxonomically identified using *Flora Iberica* (Castroviejo et al. 1986-2005) for the published families while the rest of taxa were identified according to Valdés et al. (1987) and Tutin et al. (1964–1980).

## Datasets

The original database of SINFONEVADA contains two types of information: forest attributes (and related information), and occurrence data. There are several national forest inventories (Spanish National Forest Inventory, Alberdi et al. 2010) that have partially monitored some forests of Sierra Nevada. However, due to grain size, none have been as exhaustive as the SINFONEVADA inventory.

**Figure 4.**
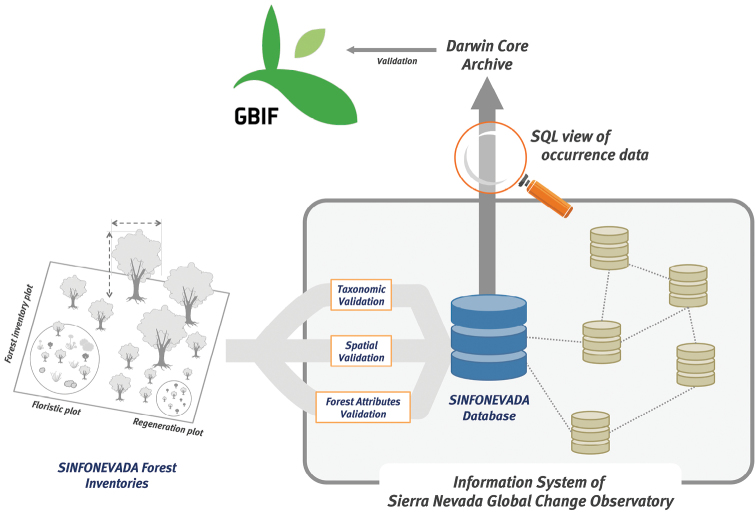
Diagram of integration of SINFONEVADA within Information System of Sierra Nevada Global Change Observatory. The original database of SINFONEVADA contains two types of information: forest attributes and related information and occurrence data. This information was integrated into the Information System of Sierra Nevada Global Change Observatory. After a validation process (see Quality Control section) the occurrence data were accommodated to Darwin Core Archive to integrate in GBIF.

The original SINFONEVADA database was incorporated into the Information System of Sierra Nevada Global Change Observatory (Figure 4). Taxonomic and spatial validations were made on this database. Also, we carried out quality control procedures for forest attributes (detection of atypical values). A custom-made SQL view of the original SINFONEVADA was performed to gather occurrence data. The view shows occurrence data collected in the floristic inventories associated with the forest inventory. We included only records that had been accepted for publication. The occurrence data were accommodated to the Darwin Core Archive to integrate in GBIF. We used Darwin Core Archive Validator tool (http://tools.gbif.org/dwca-validator/) to check whether the dataset meets Darwin Core specifications. The Integrated Publishing Toolkit (IPT v2.0.5) of the Spanish node of the Global Biodiversity Information Facility (GBIF) (http://www.gbif.es:8080/ipt) was used both to upload the Darwin Core Archive and to fill out the metadata. Information about forest attributes included in the original SINFONEVADA database is available upon request.

**Figure 5.**
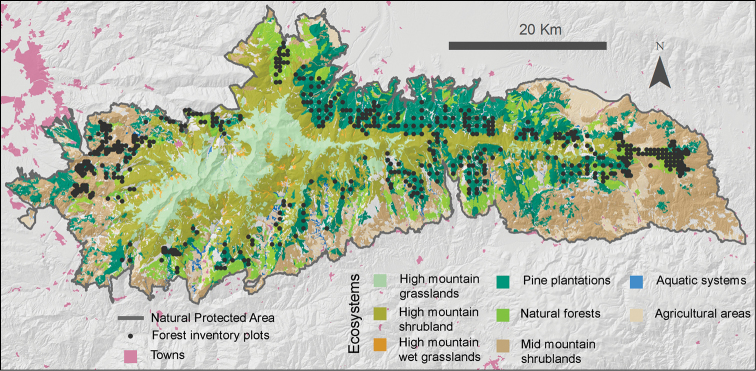
Location of the forest-inventory plots. This map shows the location of the forest-inventory plots and the distribution of the ecosystem types present in Sierra Nevada. The vegetation is predominantly high-mountain shrublands and pine plantations, with some natural forests (oaks, Pyrenean oaks, maples, etc.).

The fields provided by the SINFONEVADA dataset are:

occurrenceId, modified, basisOfRecord, institutionCode, collectionCode, catalogNumber, occurrenceRemarks, scientificName, kingdom, phylum, class, order, family, genus, specificEpithet, infraspecificEpithet, scientificNameAuthorship, continent, country, stateProvince, county, locality, minimumElevationInMeters, maximumElevationInMeters, recordedBy, identifiedBy, dateIdentified, decimalLongitude, decimalLatitude, coordinateUncertaintyinMeters.

The SINFONEVADA dataset represents an exhaustive floristic inventory of diversity of Sierra Nevada forest. It includes occurrences of 270 taxa, of which 24 are considered threatened and 9 endemic (Table 2). Information of SINFONEVADA has been used for the Natural Resources Ordinance Plan of the Sierra Nevada Natural Area (CMA 2011). Also its information provides valuable support to natural-resource managers in their decision making. It is being considered for management actions within strategies of diversification and naturalization of forests in the Sierra Nevada natural area.

## Dataset description

**Object name:** Darwin Core Archive Sinfonevada: Dataset of floristic diversity in Sierra Nevada forest (SE Spain)

**Character encoding:** UTF-8.

**Format name:** Darwin Core Archive format.

**Format version:** 1.0

**Distribution:** http://www.gbif.es:8080/ipt/archive.do?r=sinfonevada

**Publication date of data:** 2013-09-24

**Language:** English.

**Licenses of use:** The “Sinfonevada: Dataset of floristic diversity in Sierra Nevada forest (SE Spain)” dataset is made available under the Open Data Commons Attribution License: http://www.opendatacommons.org/licenses/by/1.0/.

**Metadata language:** English.

**Date of metadata creation:** 2013-06-18

**Hierarchy level:** Dataset.
